# Increased Epicardial Adipose Tissue (EAT), Left Coronary Artery Plaque Morphology, and Valvular Atherosclerosis as Risks Factors for Sudden Cardiac Death from a Forensic Perspective

**DOI:** 10.3390/diagnostics13010142

**Published:** 2023-01-01

**Authors:** Timur Hogea, Bogdan Andrei Suciu, Adrian Dumitru Ivănescu, Cosmin Carașca, Laura Chinezu, Emil Marian Arbănași, Eliza Russu, Réka Kaller, Eliza Mihaela Arbănași, Adrian Vasile Mureșan, Corina Carmen Radu

**Affiliations:** 1 Department of Forensic Medicine, George Emil Palade University of Medicine, Pharmacy, Science, and Technology of Targu Mures, 540139 Targu Mures, Romania; 2 Department of Anatomy, George Emil Palade University of Medicine, Pharmacy, Science, and Technology of Targu Mures, 540139 Targu Mures, Romania; 3 Department of Histology, George Emil Palade University of Medicine, Pharmacy, Science, and Technology of Targu Mures, 540139 Targu Mures, Romania; 4 Clinic of Vascular Surgery, Mures County Emergency Hospital, 540136 Targu Mures, Romania; 5 Doctoral School of Medicine and Pharmacy, George Emil Palade University of Medicine, Pharmacy, Sciences and Technology of Targu Mures, 540142 Targu Mures, Romania; 6 Department of Vascular Surgery, George Emil Palade University of Medicine, Pharmacy, Science, and Technology of Targu Mures, 540139 Targu Mures, Romania; 7 Faculty of Pharmacy, George Emil Palade University of Medicine, Pharmacy, Science, and Technology of Targu Mures, 540139 Targu Mures, Romania

**Keywords:** coronary artery disease, intra-abdominal fat, retrospective studies, case–control studies, autopsy, risk factors, sudden cardiac death, forensics, epicardial adipose tissue

## Abstract

**Background:** In sudden cardiac deaths (SCD), visceral adipose tissue has begun to manifest interest as a standalone cardiovascular risk factor. Studies have shown that epicardial adipose tissue can be seen as a viable marker of coronary atherosclerosis. This study aimed to evaluate, from a forensic perspective, the correlation between body mass index (BMI), heart weight, coronary and valvular atherosclerosis, left ventricular morphology, and the thickness of the epicardial adipose tissue (EAT) in sudden cardiac deaths, establishing an increased thickness of EAT as a novel risk factor. **Methods**: This is a retrospective case–control descriptive study that included 80 deaths that were autopsied, 40 sudden cardiac deaths, and 40 control cases who hanged themselves and had unknown pathologies prior to their death. In all the autopsies performed, the thickness of the epicardial adipose tissue was measured in two regions of the left coronary artery, and the left ventricular morphology, macro/microscopically quantified coronary and valvular atherosclerosis, and weight of the heart were evaluated. **Results**: This study revealed a higher age in the SCD group (58.82 ± 9.67 vs. 53.4 ± 13.00; *p* = 0.03), as well as a higher incidence in females (*p* = 0.03). In terms of heart and coronary artery characteristics, there were higher values of BMI (*p* = 0.0009), heart weight (*p* < 0.0001), EAT of the left circumflex artery (LCx) (*p* < 0.0001), and EAT of the left anterior descending artery (LAD) (*p* < 0.0001). In the multivariate analysis, a high baseline value of BMI (OR: 4.05; *p* = 0.004), heart weight (OR: 5.47; *p* < 0.001), EAT LCx (OR: 23.72; *p* < 0.001), and EAT LAD (OR: 21.07; *p* < 0.001) were strong independent predictors of SCD. Moreover, age over 55 years (OR: 2.53; *p* = 0.045), type Vb plaque (OR: 17.19; *p* < 0.001), mild valvular atherosclerosis (OR: 4.88; *p* = 0.002), and moderate left ventricle dilatation (OR: 16.71; *p* = 0.008) all act as predictors of SCD. **Conclusions**: The data of this research revealed that higher baseline values of BMI, heart weight, EAT LCx, and EAT LAD highly predict SCD. Furthermore, age above 55 years, type Vb plaque, mild valvular atherosclerosis, and left ventricle dilatation were all risk factors for SCD.

## 1. Introduction

Sudden cardiac death (SCD) still represents an enormous public health problem, ranked by WHO as the top global cause of death with over 16% of the world`s total deaths [1]. The adult population has the highest incidence of SCD, with males being at a greater risk [2,3]. Obesity (BMI > 30 kg/m^2^) and cardiovascular diseases, including coronary heart disease, atrial fibrillation, and hypertension [3,4,5,6,7], are also included as risk factors.

From the forensic perspective, it represents the most frequent manifestation of an unknown cardiovascular pathology defined by an abrupt loss of consciousness, with or without preceding symptoms, with death occurring in under an hour to a relatively healthy individual or one with undiagnosed cardiovascular pathologies. This sudden unexpected death raises suspicions in the justice system; by Romanian law, an autopsy is mandatory. Even if the death occurred at home, in most cases, the medical examiner does not have in his/her records any pathology that could be noted as the cause of death, making it a forensic case. Epicardial adipose tissue (EAT) is now considered an actual visceral fat, with characteristics of a high insulin-resistant tissue, and has a significant function in the release of proinflammatory cytokines such as TNF-alfa, IL-6, IL-7, IL-8, and macrophage accumulation [8,9]. Increased EAT thickness and volume may be an independent risk factor for coronary atherosclerosis, atrial fibrillation, and possibly left ventricular cardiomyopathy, all of which are known risk factors for SCD [9,10,11,12]. Anatomically, EAT has direct contact with the myocardium and coronary arteries, without any type of fascia interposed between them, all being nourished by the same micro-vascularization provided by the coronary arteries [13,14,15]. EAT bioactive molecules, including the abovementioned proinflammatory cytokines, are paracrinally or vasocrinally exchanged in the myocardium and the coronary artery wall, influencing their physiology [16,17,18,19,20]. By introducing EAT as a novel standalone risk factor for SCD, it can be easily evaluated with radiological investigations, with a higher thickness indicating the need for additional non-invasive and invasive cardiovascular examinations.

This study aims to demonstrate the predictive impacts of body mass index (BMI), heart weight, EAT of the left circumflex artery (LCx), EAT of the left anterior descending artery (LAD), valvular atherosclerosis, and left ventricle dilatation on the risk of SCD, from a forensic perspective.

## 2. Materials and Methods

### 2.1. Study Design

For this retrospective case–control study, 80 autopsies performed at the Institute of Legal Medicine, Targu Mures, Romania, in the year 2020 were chosen (Caucasians, 16 females, 64 males; 13–80 years old, 13.8–35.8 BMI), splitting them into a group of 40 consecutive SCD cases and a control group of 40 consecutive deaths with unknown pathologies of people who hanged themselves (A–H).

### 2.2. Sudden Cardiac Death Group

The SCD cases were identified, according to Romania’s penal law code, as those who died suddenly in apparent good health (with no prior known cardiovascular pathologies) within 24 h of the onset of any symptoms (if they were present), with or without witnesses, regardless of where the deaths had occurred or if resuscitation/intensive care measures were applied, excluding violent deaths where the toxicology report revealed the presence of alcohol, drugs, illegal substances, poison, or other chemical compounds in lethal doses. Cases found in an advanced state of putrefaction at home were also excluded.

### 2.3. Control Group

The control group was selected from a series of violent hanging deaths that could have presented cardiovascular pathologies, but the cause of their death was clear and unrelated to those, and the cardiovascular systems remained intact for optimal evaluation.

### 2.4. Heart Characteristics and Left Coronary Artery Plaque Morphology

**Figure 1:**
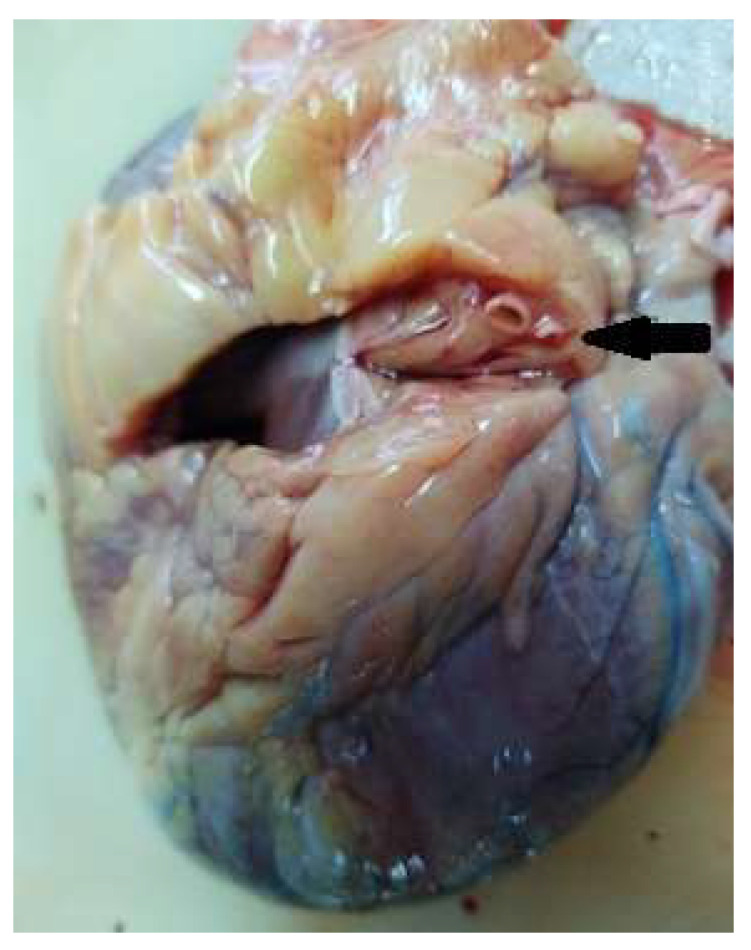
EAT thickness for Cx incision.

**Figure 2:**
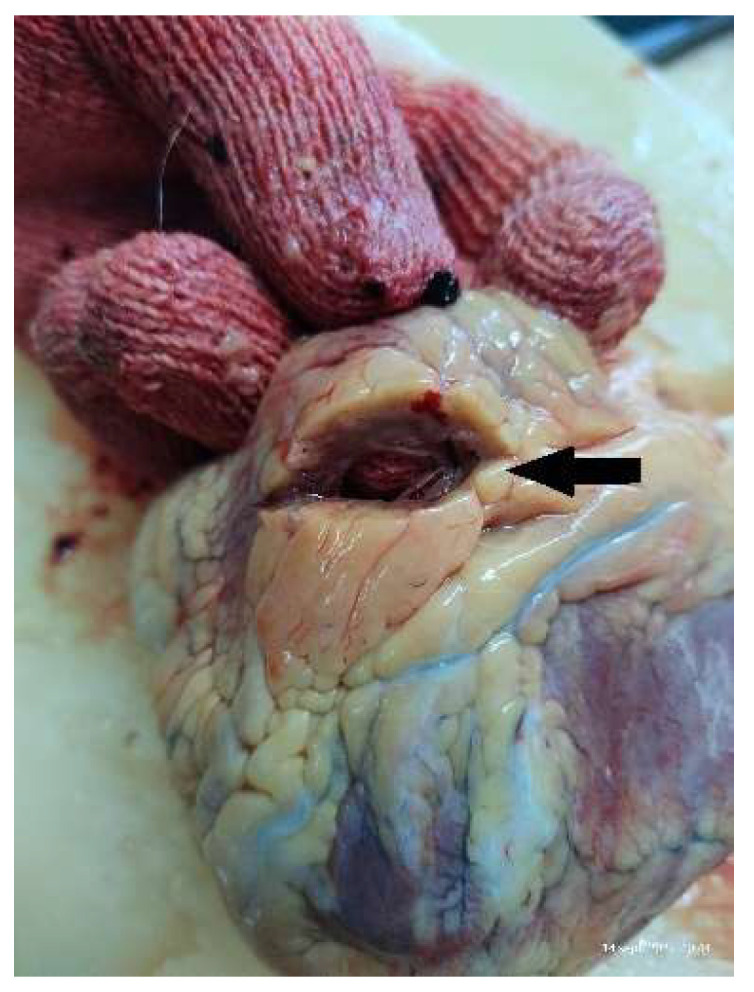
EAT thickness for LAD incision.

The histopathological examinations were performed on cardiovascular tissue fragments fixed in 10% formaldehyde for at least 24 h, with paraffin sections colored using standard hematoxylin/eosin and examined by the pathologist. The macroscopic cardiovascular pathological findings were evaluated during the autopsies by two independent forensic doctors. In all autopsies performed, we measured the thickness of the left ventricle (LV) EAT (measured at the two left coronary branches) (Figure 1 and Figure 2), the LV morphology (hypertrophy and dilatation), and weighed the heart. Valvular atherosclerosis was macroscopically rated using the following scale: absent (no deposits), mild (<3 mm spot-like deposits), moderate (>3 mm deposits ± calcifications), and severe (extensive calcification with mobility loss) [21].

The severity of LV dilatation was determined using the American Society of Echocardiography criteria based on the LV internal diameter [22]: normal (42 to 59 mm for males; 39 to 53 mm for females), mildly dilated (60 to 63 mm for males; 54 to 57 mm for females), moderately dilated (64 to 68 mm for males; 58 to 61 mm for females), and severely dilated (>68 mm). The internal diameter of the LV was then measured on the mid-ventricular transverse section. Furthermore, the enrollment was based on our assessment of the subject in terms of the abovementioned levels of severity.

The final extent of coronary atherosclerosis was determined after the histopathological examination that followed the autopsy, according to the histological grading of the American Heart Association Committee on Vascular Lesions of the Council on Arteriosclerosis, as follows: initial lesion (type I), progression-prone and resistant lesion (type II), pre-atheroma (intermediate lesion) (type III), atheroma (type IV), fibroatheroma (type Va), calcific lesion (type Vb), fibrotic lesion (type Vc), and lesion with the presence of a surface defect, hematoma, hemorrhage, or thrombotic deposit (type VI) [23].

### 2.5. Statistical Analysis

SPSS for Mac OS version 28.0.1.0 was used for the statistical analysis (SPSS, Inc., Chicago, IL, USA). Chi-square tests were used to assess the associations of the ratios with the category factors, while Student t- or Mann–Whitney tests were used to assess differences in the continuous variables. To analyze the predictive power and to establish the cut-off values of BMI, heart weight, EAT LCx, and EAT LAD, the receiver operating characteristic (ROC) curve analysis was utilized. The ROC curve analysis was used to determine the appropriate BMI, heart weight, EAT LCx, and EAT LAD cut-off values based on the Youden’s index (Youden’s Index = Sensitivity + Specificity − 1, ranging from 0 to 1). To identify independent predictors of sudden cardiac death, a multivariate logistic regression analysis using variables with *p* < 0.1 was undertaken.

## 3. Results

For the 80 autopsies included in this study, a higher age was found in the SCD group 58.82 ± 9.67 vs. 53.4 ± 13.00 (*p* = 0.03), as well as a higher incidence of females (*p* = 0.03) and higher BMI (*p* = 0.0009). In terms of the heart and coronary artery characteristics, higher values of heart weight (*p* < 0.0001), EAT LCx (*p* < 0.0001), and EAT LAD (*p* < 0.0001) were observed. Moreover, regarding the histological type of coronary artery plaque, in the A–H group there was a higher incidence of no lesion (*p* = 0.01) and type I plaque (*p* = 0.002). In contrast, in the SCD group there was a higher incidence of type Vb (*p* = 0.003). In addition, higher incidences of no valvular atherosclerotic lesion in the A–H group (*p* = 0.0001) and no LV dilatation (*p* = 0.001) were reported, as well as higher incidences of mild valvular atherosclerotic lesion (*p* = 0.001) and moderate LV dilatation (*p* = 0.008). The rest of the characteristics are shown in Table 1.

**Figure 3:**
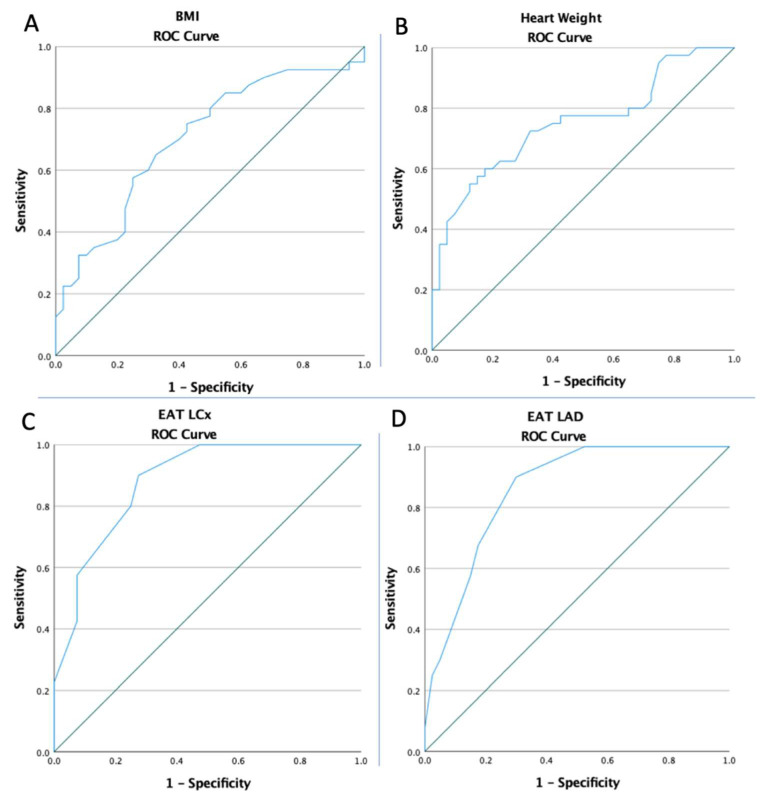
The ROC curve analyses concerning SCD (**A**) for the BMI (AUC: 0.703; *p* = 0.002), (**B**) for the heart weight (AUC: 0.750; *p* < 0.0001), (**C**) for the EAT LCx (AUC: 0.881; *p* < 0.0001), and (**D**) for the EAT LAD (AUC: 0.857; *p* < 0.0001).

The ROC curves of BMI, heart weight, EAT LCx, and EAT LAD were created to determine whether the baselines of these markers predicted SCD (Figure 3). Each optimal cut-off value was obtained from the Youden’s index, the area under the curve (AUC), and the prediction accuracies of the markers are listed in Table 2.

A multivariate analysis was used to determine the association between the BMI, heart weight, EAT LCx, EAT LAD, histological type of left coronary artery plaque, valvular atherosclerosis, and left ventricle dilatation, and the risk of SCD. As a result, high baseline values of BMI (OR: 4.05; *p* = 0.004), heart weight (OR: 5.47; *p* < 0.001), EAT LCx (OR: 23.72; *p* < 0.001), and EAT LAD (OR: 21.07; *p* < 0.001) were strong independent predictors of SCD. Moreover, as shown in Table 3, age beyond 55 years (OR: 2.53; *p* = 0.045), type Vb plaque (OR:17.19; *p* < 0.001), mild valvular atherosclerosis (OR: 4.88; *p* = 0.002), and moderate left ventricle dilatation (OR: 16.71; *p* = 0.008) act as predictors of SCD. Furthermore, male sex (OR: 0.25; *p* = 0.03), no lesion (OR: 0.06; *p* = 0.01) or type I plaque (OR: 0.08; *p* = 0.002), no valvular atherosclerosis (OR: 0.13; *p* < 0.001), and no left ventricle dilatation (OR: 0.19; *p* < 0.001) act as protective factors against SCD as seen in Table 3.

## 4. Discussions

Although we acknowledge the difficulty in determining the precise number of SCD cases in any given micro/macro-region, we cannot negate that it remains a large epidemiological issue that requires serious interdisciplinary attention in order to identify any possible leads that may contribute to a solution.

This study’s key finding is that high baseline EAT values at the LCx and LAD levels, as well as BMI, heart weight, moderate LV dilatation, mild valvular atherosclerosis, age beyond 55, and coronary artery plaque morphology, are all independent predictors of SCD. Additionally, male sex, the absence of valvular atherosclerotic damage, and the absence of left ventricle dilatation all act as protective factors against SCD.

An important finding is that moderate valvular atherosclerotic and severe left ventricle dilatation are not predictors of SCD, as observed in the multivariate analysis. This aspect can be explained by the existence of only four autopsies with mild valvular atherosclerotic damage and five autopsies with significant dilatation of the left ventricle. Moreover, it is well known that male sex is a risk factor for SCD. As we see in Table 1, there 70% of males are in the SCD group, but in the control group, we have a higher incidence of males committing suicide, but we consider this is just isolated data. A future paper will be conducted to analyze the sex-specific characteristic and autopsy markers with predictive roles in SCD.

The most important finding of our study is that a significant percentage of SCD victims have unknown cardiovascular diseases (silent myocardial infarction, advanced coronary disease, cardiomyopathies) that do not manifest any symptoms, remain undiagnosed and untreated throughout their lifespan, and are only discovered at autopsy [24,25].

In developed countries, a key modifiable risk factor is obesity [26]. Several studies recently published in the literature have revealed an association between higher BMI/obesity and the risk of SCD [27,28,29,30,31,32,33]. In a study of 3,684 subjects with SCD, Finocchiaro et al. [29] found that in the case of obese sudden cardiac subjects, the incidences of LV hypertrophy (12% vs. 2%; *p* < 0.001) and critical coronary artery disease (12% vs. 3%; *p* < 0.001) were higher. In a multivariate analysis, Andersson et al. [31] found that diabetes (OR: 1.83; 95% CI: 1/30–2.59), high BMI (OR: 1.05; 95% CI: 1.02–1.08), and male sex (OR: 1.42; 95% CI: 1.001–2.01) were all associated with SCD. Furthermore, the role of LV morphology in SCD risk has been extensively studied [34,35,36,37,38,39,40,41]. According to Narayanan et al. [35], the presences of moderate or severe LD dilatation (*p* = 0.001) and severe LD dilatation (*p* < 0.001) are more common in SCD patients, and severe LV dilatation (OR: 2.5; *p* = 0.04), being an independent factor of SCD, was an independent factor of SCD in the multivariate analysis. Regarding valvular atherosclerosis, Owens et al. [41] found in a cohort of 6685 participants aged 45–84 years that the presence of aortic valve atherosclerosis is a predictor of cardiovascular events (HRL 1.50; 95% CI: 1.10–2.03) and coronary ones (HR: 1.72; 95% CI: 1.19–2.49).

In addition, Wang et al. [42], discovered that the presence of calcifications at the level of cardiac valves is an independent factor for all-cause mortality (HR: 2.50; 95% CI: 1.32–4.76; *p* = 0.005) and cardiovascular mortality (HR:5.39; 95% CI: 2.16–13.48; *p* = 0.0003) in 192 patients with long-term peritoneal dialysis.

Regarding coronary artery plaque morphology, Ho Yun et al. [43] reported prospective research that evaluated the risk of major adverse cardiovascular events over 3.4 years. They included 3115 patients with acute coronary syndrome who underwent percutaneous coronary intervention with three-vessel grayscale and virtual histology intravascular ultrasound and found that severe stenosis (>70%) and coronary plaque thin-cap fibroatheroma were associated with an increased risk of major adverse cardiovascular events (*p* < 0.0001).

Visceral adipose tissue, which recently included EAT, has an active endocrine and paracrine role by releasing several bioactive molecules (adipokines) that influence BMI, inflammation, atherosclerosis (coronary and valvular), and diastolic dysfunction, leading to cardiovascular diseases and heart failure [44,45]. In SCD studies, EAT has gained special interest because of its intimal relation with both coronary arteries and the myocardium, which is related to insulin resistance, ventricular mass, low-density cholesterol levels, and arterial blood pressure [46,47]. Eroglu S et al., through 2D echocardiography and coronary angiography, showed that EAT thickness can be used as a potential marker for the presence of coronary artery disease [48]. Following a thorough literature review, Steven et al. [49] concluded that higher left ventricular mass can be a predictor for SCD; however, our investigation found no significance regarding this matter. Haider et al. [50] analyzed 3661 participants of the Framingham Heart Study [51] and demonstrated that left ventricular hypertrophy, defined as >143 g/m in males and >102 g/m in females, was present in 21.5% of SCD cases, and for every 50 g increase in LV mass there was a 1.45-fold increased risk of SCD. EAT prospective studies concluded that high EAT volumes measured radiologically were independently associated with a high risk of SCD after 6.1 years of follow-up. They also included EAT among established cardiovascular disease risk prediction variables [52]. A post hoc analysis in the CRISP CT study found that the epicardial fat attenuation index, which marks coronary inflammatory load, was predictive and raised the risk prediction algorithms in cardiac mortality [53]. Regarding the morphology of atherosclerotic plaque, Niculescu et al. [54] found unstable plaques (OR: 2.83; *p* < 0.001 and OR: 2.40; *p* = 0.04) as independent predictors of restenosis and mortality in 369 patients with carotid endarterectomy.

As our postmortem findings concluded, Levelt et al. [55] also found that alive patients with lower BMIs had lower volumes of EAT and normal cardiac function, signaling that EAT could be a factor in mediating cardiac dysfunction, in particular LV mass and volume. Most studies that evaluated the volume of EAT as a cardiovascular risk factor were conducted using radiological methods [56] on living patients (echocardiography, CT, or cardiac magnetic resonance imaging). Unknown myocardial scars identified using MRI during screening procedures may be more accurate and specific than a low ejection fraction in identifying high-risk patients that are candidates for internal cardiac defibrillators [38,57]. PET-CT 18F FDG or a gallium scan has proved useful in detecting cardiac sarcoidosis or myocarditis, which are potential risk factors for SCD. For the young male population or athletes of both sexes, a cardiac CT scan may detect coronary atherosclerosis or hereditary anomalies (coronary bridging) that can lead to an SCD event during intense physical activity. Echocardiography measurements of EAT have their limitations, can be interpreted as pericardial effusion [58], can be confused with pericardial adipose tissue, or the small acoustic window used can create measurement problems for an unexperienced examiner, providing just a rough estimation of the EAT volume. CT, MRI, and proton spectroscopy methods are considered gold standards in developed countries, but these volume measurements are only made for patients already diagnosed with cardiovascular pathologies [59,60]. Focusing on prophylactic measures, alongside exercise training, recent studies showed that EAT volume can also be reduced by GLP-1 analogs and SLGT2 inhibitors [61,62,63,64], with direct effects of reducing systemic inflammation, coronary atherosclerosis, and myocardial damage, all of which are established risk factors for SCD.

We found very few studies that implied direct measurements through autopsy findings. According to Risgaard et al. [65], establishing a postmortem etiology for a nationwide study of SCD autopsy reports of individuals below 50 years of age remained unsuccessful for up to 31% of cases. Although not implemented in Romania yet, postmortem imaging investigations will provide a strong complementary tool to the conventional autopsy. Postmortem cardiac magnetic resonance imaging will offer a better understanding of the cardiovascular diseases responsible for SCD [66].

In an attempt to reduce the incidence of SCD in the general population, studies have prioritized selecting the cases that survived an acute cardiovascular event, but few studies marked autopsy-related, histopathological, or genetic findings. In this perspective, based on the US Framingham Risk Score and Europe-based HeartScore [67] for estimating cardiovascular risks, treatment protocols were established and, to some degrees, were successful in statistically lowering the incidence of SCD, but still, the elephant in the room remained unnoticed. The great majority of SCD cases have no earlier signs or symptoms until the fatal event occurs, indicating undiscovered and untreated cardiovascular pathologies, most commonly belonging to the active population that seems to be in good physical health, with sudden death remaining the first and sole event. In recent years, there has been a shift from SCD cases belonging to the high-risk group to cases belonging to larger subgroups that are at lower risk, making it difficult to achieve better risk stratification and uncover new risk factors in an attempt to reduce the mortality rate [68].

## 5. Conclusions

Our data revealed that higher baseline values of BMI, heart weight, EAT LCx, and EAT LAD highly predict SCD. Furthermore, age above 55, type Vb plaque, mild valvular atherosclerosis, and left ventricle dilatation were risk factors for SCD. In perspective, a higher BMI assessment could signal the need for an EAT thickness radiological measurement, an increase in it making the person a candidate for an echocardiogram and a coronary angiography evaluation. These preventative measures will reduce the occurrence of SCD, at least for the deceased’s living relatives, keeping in perspective the inclusion of larger subgroups at low risk from which most cases come.

## Figures and Tables

**Table 1:** All characteristics regarding the demographic, heart, valvular, and coronary artery of all the autopsies that were included.

Variables	All Autopsies *N* = 80	A–H *n* = 40	SCD *n* = 40	*p*-Value (OR; CI 95%)
Age (years) MEAN ± SD	56.11 ± 11.7	53.4 ± 13.00	58.82 ± 9.67	0.03
Male/female (sex) no. (%)	64 (80%) 16 (20%)	36 (90%) 4 (10%)	28 (70%) 12 (30%)	0.03 (0.25; 0.07–0.89)
**Heart and Coronary Artery Characteristic**				
BMI (kg/m^2^), median [Q1–Q3]	24.8 [23.25–26.6]	23.9 [22.35–25.5]	25.8 [24.42–28.2]	0.0009
Heart weight (g), median [Q1–Q3]	420 [368.75–485]	390 [347.5–425]	480 [407.5–550]	<0.0001
EAT LCx (cm), median [Q1–Q3]	0.6 [0.3–0.725]	0.3 [0.3–0.525]	0.7 [0.6–0.8]	<0.0001
EAT LAD (cm), median [Q1–Q3]	0.6 [0.5–0.8]	0.5 [0.3–0.6]	0.8 [0.6–0.925]	<0.0001
Lv thickness (cm), median [Q1–q3]	1.1 [1–1.225]	1 [1–1.3]	1.2 [1–1.2]	0.15
IV thickness (cm), median [Q1–Q3]	1.2 [1–1.3]	1.2 [1–1.3]	1.2 [1–1.425]	0.19
**Histological Type of Left Coronary Artery Plaque**				
No lesion, no. (%)	11 (13.75%)	11 (27.5%)		0.01
Type I, no. (%)	17 (21.25%)	15 (37.5%)	2 (5%)	0.002
Type II, no. (%)	4 (5%)	4 (10%)		0.12
Type III, no. (%)	6 (7.5%)	3 (7.5%)	3 (7.5%)	ns
Type IV, no. (%)	3 (3.75%)		3 (7.5%)	0.18
Type Va no. (%)	8 (10%)	5 (12.5%)	3 (7.5%)	0.46
Type Vb, no. (%)	19 (23.75%)		19 (47.5%)	0.003
Type Vc no. (%)	4 (5%)	1 (2.5%)	3 (7.5%)	0.32
Type Vi, no. (%)	8 (10%)	1 (2.5%)	7 (17.5%)	0.053
**Valvular Atherosclerosis**				
Absent, no. (%)	46 (57.5%)	32 (80%)	14 (35%)	0.0001 (0.13; 0.04–0.37)
Mild, no. (%)	30 (37.5%)	8 (20%)	22 (55%)	0.001 (4.88; 1.80–13.21)
Moderate, no. (%)	4 (5%)		4 (10%)	0.12 (9.98; 0.51–191.91)
**Left Ventricle Dilatation**				
Absent, no. (%)	33 (41.25%)	24 (60%)	9 (22.5%)	0.001 (0.19; 0.07–0.51)
Mild, no. (%)	29 (36.25%)	15 (37.5%)	14 (35%)	0.81 (0.89; 0.36–2.23)
Moderate, no. (%)	13 (16.25%)	1 (2.5%)	12 (30%)	0.008 (16.7; 2.05–136.08)
Severe, no. (%)	5 (6.25%)		5 (12.5%)	0.09 (12.5; 0.67–235.01)

**Table 2:** The AUCs of the ROC curves, 95% confidence intervals, sensitivities, and specificities of the BMI, heart weight, EAT LCx, and EAT LAD.

Variables	Cut-Off	AUC	Std. Error	95% CI	Sensitivity	Specificity	*p*-Value
	**Sudden Cardiac Death**						
BMI	25.5	0.703	0.059	0.587–0.818	57.5%	75%	0.002
Heart weight	417.5	0.750	0.055	0.643–0.858	72.5%	67.5%	<0.0001
EAT LCx	0.45	0.881	0.037	0.808–0.954	90%	72.5%	<0.0001
EAT LAD	0.55	0.857	0.042	0.775–0.940	90%	70%	<0.0001

**Table 3:** Multivariate analysis for predictors of SCD.

	Sudden Cardiac Death		
	OR	95% CI	*p*-Value
>55 years	2.53	1.02–6.29	0.045
Male sex	0.25	0.07–0.89	0.03
	**Histological Type of Left Coronary Artery Plaque**		
No lesion	0.06	0.008–0.55	0.01
Type I plaque	0.08	0.01–0.41	0.002
Type Vb plaque	17.19	3.64–81.10	<0.001
		**Valvular Atherosclerosis**	
Absent	0.13	0.04–0.37	<0.001
Mild	4.88	1.80–13.21	0.002
		**Left Ventricle Dilatation**	
Absent	0.19	0.07–0.51	<0.001
Moderate	16.71	2.05–136.07	0.008
Severe	6.88	0.78–60.06	0.08
High BMI	4.05	1.56–10.51	0.004
High heart weight	5.47	2.09–14.28	<0.001
High EAT LCx	23.72	6.83–82.36	<0.001
High EAT LAD	21.07	6.11–72.18	<0.001

## Data Availability

Not applicable.
